# The Effectiveness of Frontal Plane Adaptability in a Novel Foot Prosthesis: Protocol for a Randomized Crossover Clinical Trial

**DOI:** 10.2196/84313

**Published:** 2025-12-23

**Authors:** Stephanie L Carey, M Jason Highsmith, Saifullah Farrag, Molly C Gries, Katheryn J Allyn, Andrew T Humbert, Donald J Fogelberg, James M Colvin, Matthew M Wernke, Murray E Maitland

**Affiliations:** 1 Department of Mechanical and Aerospace Engineering, University of South Florida, 4202 E Fowler Ave, ENG 030, Tampa, FL, 33620, United States, 1 813 974 5765; 2 School of Physical Therapy and Rehabilitation Sciences, Morsani College of Medicine, University of South Florida, Tampa, FL, United States; 3 Department of Rehabilitation Medicine, University of Washington, Seattle, WA, United States; 4 WillowWood Global, LLC, Mt. Sterling, OH, United States

**Keywords:** prosthetic foot, lower extremity amputation, clinical trial, gait analysis, mixed methods

## Abstract

**Background:**

The primary objective of this study is to investigate whether a novel prosthetic foot with a polycentric ankle, which offers side-to-side adaptability, improves mobility and daily performance in individuals with (1) unilateral above-knee amputation, (2) unilateral below-knee amputation with a lower mobility level (K2) , or (3) bilateral amputation, any level of amputation. Traditional prostheses are primarily designed for forward walking and lack adaptability for navigating uneven terrain, turning, and executing other multidirectional tasks, often resulting in pain, instability, and limited function.

**Objective:**

The objective of this testing protocol is to better understand the functional impact of a novel foot design that offers more inversion/eversion rotation of the polycentric ankle. Although the foot has the potential to address mobility challenges, it is unknown whether people with different amputations and mobility levels respond differently.

**Methods:**

The study includes community-based mobility assessments, standardized questionnaires, and participant feedback. Although side-to-side adaptability prosthetic feet have shown benefits for highly active users, this study aims to evaluate the impact on individuals with more significant mobility limitations.

**Results:**

Results may guide prosthetic prescription, expand access to advanced prosthetic technology, and inform reimbursement policies, particularly benefiting veterans and service members with combat-related amputations. The study began recruiting in October 2023; as of September 2025, there were 66 participants (17 below the knee, 34 above the knee, and 15 bilateral) who completed the protocol. The clinical trial is expected to be completed by March 2027. Dissemination of results is expected to be completed by December 2027.

**Conclusions:**

This procedural study presents a model for evaluating prosthetic feet and other mobility-assistive technologies in real-world contexts, supporting broader efforts to match components to user needs through clinical trials that are grounded in both functional performance and user experience.

## Introduction

According to 2005 projections, over 1 million adults in the United States live with lower extremity amputation (LEA) [1], with approximately 22,000 people undergoing above-knee amputations annually [2]. Additionally, about 12,000 individuals have bilateral amputations, though precise figures are unavailable [1]. The largest group of individuals seen by prosthetists consists of those with transtibial amputations and limited mobility, classified at Medicare Functional Classification Level (MFCL) K2, accounting for 36% of the lower extremity amputee population [3]. A person with LEA at MFCL K2 can use a prosthesis for transfers and ambulation on level surfaces, with the potential to walk in environments with low-level barriers such as curbs, stairs, or uneven surfaces.

Advancements in prosthetic technology have significantly improved the mobility and quality of life for individuals with LEAs. However, the challenge remains in providing tailored prosthetic solutions that optimize both functional outcomes and personal satisfaction for diverse patient populations, especially those with unique needs such as warfighters, veterans, and individuals with bilateral or above-knee amputations. The importance of patient-specific prescriptions cannot be overstated, as prosthetic components must meet the specific biomechanical requirements of each individual to maximize effectiveness.

**Figure 1.**
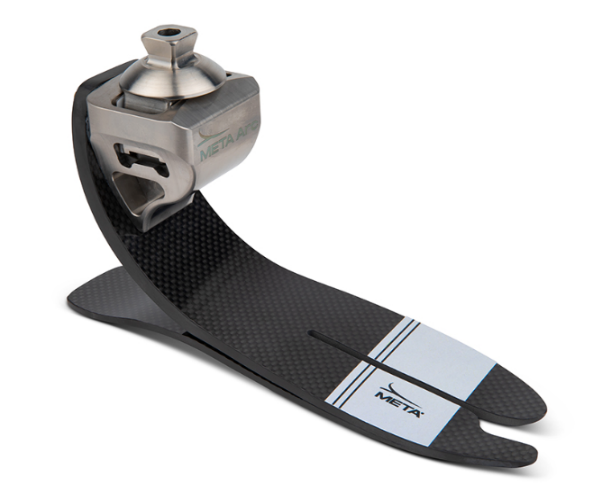
META Arc (WillowWood).

The need for innovative solutions is particularly pressing for individuals with higher levels of amputation and those experiencing lower levels of mobility. For example, individuals with bilateral amputations face persistent difficulties with balance, cognitive focus during gait, overall mobility, and residual limb pain despite using conventional prosthetic devices. Recent developments in prosthetic ankle technology have shown promising improvements, particularly in enhancing the foot’s adaptability to different surfaces, which is crucial for optimizing function in various environments. The META Arc (WillowWood), shown in Figure 1, features 10 degrees of frontal plane motion, which may enhance mobility, balance, and function during activities that benefit from this additional motion, such as turning, side steps, and backward steps.

In our previous study, 21 participants with unilateral below-knee amputation had 16 models of usual prosthetic feet, making comparisons between the usual foot and an investigational foot challenging [4]. Also, comparisons with the usual foot do not isolate the benefits of the unique features of the investigational foot. In the previous study, a locking feature eliminated the mediolateral motion, rendering the foot comparable to a typical energy storage and return foot. This was only completed for gait testing protocols in the laboratory; participants stated they had no opportunity for community testing of the locked version. The current study attempts to overcome some of these limitations.

This paper outlines the procedural approach for a randomized clinical trial designed to evaluate the impact of a novel frontal plane ankle technology on individuals with above-knee or bilateral amputations or those with below-knee amputations classified at an MFCL K2. By using a double crossover design and focusing on person-centered outcomes, this study seeks to improve our understanding of how advanced prosthetic feet can enhance functional mobility, balance, and daily activity participation for individuals with complex amputation profiles. A two-period or double crossover research design provides more data per participant, balances potential carryover effects, and improves statistical power and precision by having each participant’s response with the usual prosthetic foot, with the novel foot in both the locked and unlocked position, and with the usual prosthetic again. The mixed methods approach used in this study, which incorporates qualitative insights from interviews, daily experiences recorded in logbooks, and surveys, along with objective measures from standard functional tests, provides a comprehensive evaluation of the novel prosthetic foot. This protocol paper can be a model for not only evaluating prosthetic components but other assistive technologies as well.

The findings from this ongoing study are expected to have far-reaching implications for clinical practice, particularly for military service members and veterans who often face unique and severe challenges related to limb loss. With more than 1 million individuals living with LEA in the United States, and a significant portion of these being veterans or military personnel, the outcomes of this research will provide valuable insights into how personalized prosthetic technology can improve long-term mobility and quality of life for this population. This study challenges prosthesis users to move sideways, move backwards, and turn. By completing well-studied clinical assessments on an instrumented walkway, this study allows for biomechanical component analysis, providing more insight into prosthesis use and design.

## Methods

### Overview

This prospective multisite randomized double crossover clinical trial study will follow an A1-B1-B2-A2 design that includes military veterans and civilians. Three collaborative sites will recruit and enroll participants: (1) University of Washington (Seattle, WA), (2) WillowWood (Mt. Sterling, OH), and (3) University of South Florida (Tampa, FL). Prior to data collection, the principal investigators and main study personnel from each site participated in a study protocol training session to ensure data collection at each site was the same. The study personnel meet every other week to discuss study procedures.

### Ethical Considerations

The study received approval (STUDY00017239) from the University of Washington’s institutional review board (IRB); the IRBs of the other sites and the US Army’s Office of Human Research Oversight reviewed the study protocol. The protocol is listed in the federal clinical trial registry (ClinicalTrials.gov NCT06214026).

All participants will provide informed consent. Study data will be confidential. There will be links between study data and participant identifiers that will be kept in separate secured areas. The link between participant identifiers and the research data will be destroyed after the records retention period required by state and/or federal law. All the information provided by the participants will be confidential.

After the completion of visits 2, 3, and 4, participants will be compensated US $200 per visit for a total of US $600. Parking, mileage, and transportation (within limits) will be paid for study visits.

### Participants

The study includes three groups of individuals with amputations: (1) people with a unilateral above-knee amputation, (2) people with bilateral LEAs, and (3) people with a unilateral below-knee amputation who are categorized as having a K2 limited community ambulation level of mobility. All participants must be able to provide written informed consent independently and have the ability to read, write, and comprehend English.

Past research studies of people with amputations have historically underrepresented specific groups. Barriers to participation in clinical trials have been identified. This study targets individuals in these categories, and accommodations have been made. These included flexible payment schedules for participation, free rideshare transportation organized by study staff, free parking for participants who had their own transportation, and an on-call prosthetist. In addition, the study enrollment period was 3 years, enabling participants to delay protocol initiation due to socket changes, skin issues, or personal factors.

### Eligibility Criteria

To be eligible for the study, participants must be 16 years or older and reside in an independent living environment within the community. They may use a walking aid but should not primarily rely on wheelchair mobility.

### Exclusion Criteria

The weight limit is 165.6 kg (365 lbs). Participants must not have conditions, such as skin wounds, that would preclude the use of a prosthesis. Young children will not be included because they have different functional needs compared to adults. People will be excluded if they have fluctuating conditions that may significantly alter gait mechanics during the 10-week study. Examples include Parkinson disease, alcoholism, brain tumors, and hereditary cerebellar ataxias.

### Discontinuation Criteria

Participants will be discontinued from the study if they are unable to use a prosthesis. Examples include surgery or trauma. However, due to the potential for fluctuations in prosthesis use among this population, short-term issues will not disqualify individuals from continued participation.

### Procedures

#### Randomization

Stratified by study site, block randomization will be used to determine the order in which participants receive the locked and unlocked investigational prosthesis. Randomization will be completed and maintained through Research Electronic Data Capture (REDCap), a secure, Health Insurance Portability and Accountability Act–compliant web application for building and managing online surveys and databases.

#### Study Procedures

##### Overview

This study uses a randomized double crossover clinical trial to evaluate a carbon fiber prosthetic foot with a frontal plane–adaptable linkage in two configurations: (1) unlocked, allowing frontal plane motion, and (2) locked, restricting frontal plane motion. When locked, the foot functions comparably to a conventional energy-storing-and-returning carbon fiber foot. A secondary aim is to compare the unlocked configuration to the participant’s usual prosthetic foot, representing the current standard of care.

The study follows an A1-B1-B2-A2 crossover design:

- A conditions involve the use of the participant’s usual prosthetic foot.
- B conditions involve the study foot with the linkage either locked or unlocked. The order of the B conditions is randomized using block randomization stratified by study site.

A custom-designed pin system is used to lock the linkage and prevent frontal plane motion when required.

##### A1 (Baseline)

At the initial visit, participants provide demographic information and complete baseline questionnaires and performance assessments. They are then trained on online data entry procedures and fitted with the study prosthesis (either locked or unlocked, depending on randomization) by a licensed prosthetist. Participants with bilateral amputations receive matched prostheses. Once fitted with the study prosthesis, participants start the 2-week accommodation period.

##### B1 (First Intervention Phase)

Participants use the first randomized configuration of the study foot for two weeks during routine community activities. Throughout this phase, they submit usage data, subjective performance ratings, and qualitative feedback via an online logbook. At the end of B1, standardized questionnaires and performance assessments are administered.

##### B2 (Second Intervention Phase)

The frontal plane linkage is adjusted to the alternate configuration (locked or unlocked), and participants continue regular use of the study prosthesis for another two weeks. Data collection procedures mirror those in B1. This phase concludes with standardized assessments and a semistructured interview to compare experiences with both foot configurations.

##### A2 (Postintervention Reflection)

Participants return to using their usual prosthetic foot for two weeks. This phase is intended to facilitate reflection on the differences between the study prosthesis (particularly in its unlocked configuration) and standard care. At the final visit, participants complete additional questionnaires and a concluding semistructured interview.

### Outcome Measures

#### Physical Performance Measures

**Figure 2.**
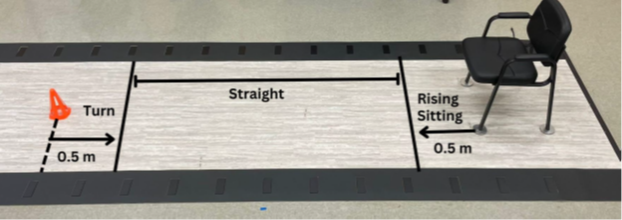
Component Timed Up and Go in the lab setting.

**Figure 3.**
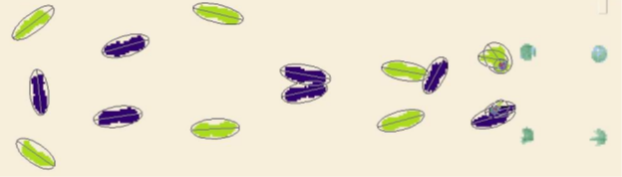
Timed Up and Go footfall patterns from the ProtoKinectics Movement Analysis Software (Zenometrics LLC).

To determine if the META Arc novel prosthetic foot frontal plane motion improves mobility, participants will be assessed with the 10-meter Walk Test [5], Timed Up and Go (TUG) [5,6], the Three Times Figure-of-Eight Walk Test (3XF8W) [7], and the Four Square Step Test [8]. In addition to the typical timed outcome of these clinically based tests, additional spatiotemporal parameters will be measured with the Zeno electronic walkway (Figure 2). Spatiotemporal parameters of gait are believed to be functionally important and may be correlated with falls [9] and corroborate benefits of increased prosthetic ankle range of motion [10]. These secondary outcomes are hypothesis-generating for this population in relation to any changes in perceived function. These secondary outcomes will include the component TUG test [11] (Figure 3) and an instrumented four square test [12].

#### Participant Qualitative Measures

The participant-related outcome measures include a demographic questionnaire and the Prosthetic Limb Users Survey of Mobility (PLUS-M). The PLUS-M is a 12-item questionnaire developed at the University of Washington Center for Outcomes Research in Rehabilitation, which examines typical everyday mobility activities on a 5-point Likert scale. Participants were also asked to fill out a logbook every day during the study. They could complete the logbook on paper or via a digital reminder system sent via email by REDCap.

Semistructured interviews will be completed during research phases B2 (investigational foot) and A2 (usual foot). These interviews will clarify significant events documented in the logbooks. The focus of interviews will be on gathering information about the participants’ experience using different prosthetic feet, with an emphasis on identifying activities that are facilitated or hindered by the functioning of the prosthetic foot.

### Statistical Analysis

The primary analysis will compare the META Arc foot in locked versus unlocked configurations. Statisticians will remain blinded to condition labels, analyzing outcomes as Condition X versus Y, with knowledge of the order (X→Y or Y→X), but not which corresponds to locked or unlocked.

Descriptive statistics (means and SDs for continuous variables; counts and percentages for categorical variables) will be used to summarize participant characteristics, stratified by amputation group. All analyses will be performed separately for each group.

### Primary Outcomes

Primary performance outcomes include the TUG, the 3XF8W, and the Four Square Step Test. Assumptions of normality and homogeneity will be tested. If met, a multivariate analysis of variance (MANOVA) will be used to evaluate the effect of foot configuration at α=.05. A permutational MANOVA will be used if assumptions are violated. If multivariate differences are significant, post hoc paired *t* tests (or Wilcoxon signed-rank tests if nonnormality is present) will be used to assess individual outcomes without correction for multiple comparisons. In order to help control the familywise type I error risk, pairwise comparisons of the primary outcomes will only occur if the multivariate difference comparison from the MANOVA is significant.

### Satisfaction Measures

Satisfaction (PLUS-M) will be analyzed using paired *t* tests (or Wilcoxon tests) at α=.025 to maintain a familywise error rate of .05 across satisfaction outcomes.

### Power

With n=30 for the above-knee and K2 groups, the study is powered (>80%) to detect a standardized effect size of 0.53. In the bilateral group (n=16), the power is greater than 50% for the same effect size. Preliminary data show larger effects (eg, 0.98 for the 3XF8W), supporting adequate power. Although underpowered to detect a meaningful difference in the bilateral group, 16 subjects is in line with other gait studies that have published data on 10 [13], 15 [14], or 19 [15] subjects with bilateral amputations, a challenging population to recruit and enroll. The study is also powered (81%) to detect a 75% preference for one configuration (vs 50% under the null) using a sign test with n=30. Although no minimal clinically important difference has been established, it is predicted that a standardized effect of at least 0.50 will correspond to an impactful improvement in participants’ physical function.

### Secondary Outcomes

Secondary outcomes (eg, 3XF8W step count, 10-meter walk, and PLUS-M subscales) will be analyzed using paired *t* tests or Wilcoxon tests at α=.05. Carryover effects will be assessed and, if present, accounted for in sensitivity analyses. As no multiple comparison corrections will be used for secondary analyses, resulting in a higher familywise type I error rate, all secondary results will be considered exploratory and hypothesis generating rather than confirmatory.

### Logbook and Walkway Data

Logbook variables (eg, perceived support on challenging surfaces) will be compared using paired *t* tests or Wilcoxon tests. Fall counts will be analyzed with a sign test. Gait variables (eg, step width, double support time) from the Zeno walkway will be analyzed descriptively.

### Qualitative Analysis

Interviews will be transcribed via Microsoft Teams or Zoom (Zoom Communications Inc) and analyzed using an inductive thematic approach. Open coding will identify themes, followed by focused coding to group codes into broader categories. Multiple codes may apply to individual responses. Qualitative analysis will follow current standards for rigor and reporting.

## Results

This study protocol, funded by the Department of Defense Orthotics and Prosthetics Outcome Program (OP220033), was approved by the University of Washington’s IRB, the University of South Florida’s IRB, and the US Army’s Office of Human Research Oversight in March of 2023. As of September 2025, a total of 66 subjects have completed the study, including 17 with below-the-knee prostheses, 34 with above-the-knee prostheses, and 15 bilateral prosthesis users. The recruitment and data collection period of the study is expected to be completed by March 2027. Dissemination of results is expected to be completed by December 2027.

It is expected that the side-by-side adaptability of the investigational prosthetic foot will improve community functions such as gait over uneven ground and cross slopes, weight shift during two-legged standing, side-stepping, turning, and precise positioning of the foot at initial stance. Evidence from this study is intended to help clinicians weigh options regarding the use of a prosthetic foot with a passive adaptable ankle in the frontal plane for their patients.

## Discussion

### Expected Findings

It is expected that the side-to-side adaptability of the META Arc prosthetic foot will help people with above-knee amputations, people with bilateral amputations, and people with lower levels of mobility manage their everyday activities better. It is expected that the results of this study will demonstrate that people with lower levels of mobility can complete non–straight gait tasks such as turning better and will participate in more challenging activities every day.

It is hypothesized that participants with bilateral amputations will report greater satisfaction, mobility, and comfort when using a prosthetic foot with frontal plane adaptability (unlocked condition) than when wearing a prosthetic foot without frontal plane adaptability (locked), as measured by the PLUS-M. It is also expected that participants will demonstrate improved performance during the 3XF8W using a prosthetic foot with frontal plane adaptability compared to a foot without frontal plane adaptability.

A cutoff for the TUG in determining fall risk of ≥8.17 seconds has been previously determined by Sawers and Hafner [16]. However, it is not clear what part of the TUG is causing the increased risk. By completing component analysis, this study will determine what part of the TUG is causing times above this fall risk threshold.

A previous study of the META Arc foot showed that optimal stiffness for walking on flat surfaces is not the same as for turning [17]. Preliminary findings have shown that there are differences in stride length and swing time when comparing turning with the prosthetic foot on the inside or the outside of the turn. This may have some implications in the development of gait training and fall prevention strategies.

During logbook and interview analysis, it is expected that participants will report their perceived changes in person-specific mobility during activities that require frontal plane adaptability. The logbook and interviews will also provide feedback on the investigational foot regarding what aspects the participants did or did not like. This information will be very useful for making improvements for the next version if needed. Preliminary logbook analysis has shown that participants have engaged in walking on uneven terrain and participated in activities such as walking the dog, hiking, and shopping with benefits while wearing the investigational foot.

The study populations are particularly relevant to veterans and service members with amputations resulting from combat-related trauma or disease. Prosthesis users should be challenged to move sideways, move backward, and turn. Biomechanical components are often conflated with typical summary scores, which are typically based on the time it takes to complete a task. By conducting component analysis, mobility challenges, prosthetic design, and training can be addressed to better fit the needs of the users.

There are some limitations to the protocol. The participants have a 2-week accommodation period for each phase of the A1-B1-B2-A2 crossover design. There is no structured training program. Although the order of locked and unlocked use of the study foot will be varied between individuals, participants may continue to become more accustomed to using the foot into the next period, with the possibility of bias in the results.

### Conclusion

The study will use a design that allows participants to get used to using two forms of the investigational foot: the locked and unlocked versions. This unique study design feature allows for comparing the investigational foot to a standardized control foot in a randomized order. Accommodation for participants in understudied groups may facilitate recruitment. Findings from this study are expected to generate new knowledge on the functional benefits of frontal plane adaptability, informing prosthetic design and clinical practice.

## Supplementary material

10.2196/84313 Peer Review Report 1 Peer review report by the Fiscal Year 2022 Orthotics and Prosthetics Outcomes Research Program, Clinical Trial Review Committee, U.S. Army Medical Research and Development Command Congressionally Directed Medical Research Programs (Department of Defense, USA).

## Abbreviations

3XF8W: Three Times Figure-of-Eight Walk Test
IRB: institutional review board
LEA: lower extremity amputation
MANOVA: multivariate analysis of variance
MFCL: Medicare Functional Classification Level
PLUS-M: Prosthetic Limb Users Survey of Mobility
REDCap: Research Electronic Data Capture
TUG: Timed Up and Go

## References

[1] Ziegler-Graham K, MacKenzie EJ, Ephraim PL, Travison TG, Brookmeyer R (2008). Estimating the prevalence of limb loss in the United States: 2005 to 2050. Arch Phys Med Rehabil.

[2] George J, Navale SM, Nageeb EM (2018). Etiology of above-knee amputations in the United States: is periprosthetic joint infection an emerging cause?. Clin Orthop Relat Res.

[3] (2015). Practice analysis of certified practitioners in the disciplines of orthotics and prosthetics. American Board for Certification in Orthotics Prosthetics & Pedorthics Inc.

[4] Maitland ME, Imsdahl SI, Fogelberg DJ (2024). Motion analysis of a frontal plane adaptable prosthetic foot. J Prosthet Orthot.

[5] Podsiadlo D, Richardson S (1991). The timed “Up & Go”: a test of basic functional mobility for frail elderly persons. J American Geriatrics Society.

[6] Ng SS, Hui-Chan CW (2005). The timed up & go test: its reliability and association with lower-limb impairments and locomotor capacities in people with chronic stroke. Arch Phys Med Rehabil.

[7] Barry E, Galvin R, Keogh C, Horgan F, Fahey T (2014). Is the Timed Up and Go test a useful predictor of risk of falls in community dwelling older adults: a systematic review and meta-analysis. BMC Geriatr.

[8] Hess RJ, Brach JS, Piva SR, VanSwearingen JM (2010). Walking skill can be assessed in older adults: validity of the Figure-of-8 Walk Test. Phys Ther.

[9] Dite W, Temple VA (2002). A clinical test of stepping and change of direction to identify multiple falling older adults. Arch Phys Med Rehabil.

[10] Heitzmann DWW, Salami F, De Asha AR (2018). Benefits of an increased prosthetic ankle range of motion for individuals with a trans-tibial amputation walking with a new prosthetic foot. Gait Posture.

[11] Clemens SM, Gailey RS, Bennett CL, Pasquina PF, Kirk-Sanchez NJ, Gaunaurd IA (2018). The Component Timed-Up-and-Go test: the utility and psychometric properties of using a mobile application to determine prosthetic mobility in people with lower limb amputations. Clin Rehabil.

[12] Gouelle A, Highsmith MJ (2020). Instrumented Four Square Step Test in adults with transfemoral amputation: test-retest reliability and discriminant validity between two types of microprocessor knees. Sensors (Basel).

[13] Major MJ, Stine RL, Gard SA (2013). The effects of walking speed and prosthetic ankle adapters on upper extremity dynamics and stability-related parameters in bilateral transtibial amputee gait. Gait Posture.

[14] Akarsu S, Tekin L, Safaz I, Göktepe AS, Yazicioğlu K (2013). Quality of life and functionality after lower limb amputations: comparison between uni- vs. bilateral amputee patients. Prosthet Orthot Int.

[15] Su PF, Gard SA, Lipschutz RD, Kuiken TA (2007). Gait characteristics of persons with bilateral transtibial amputations. J Rehabil Res Dev.

[16] Sawers A, Hafner BJ (2020). Using clinical balance tests to assess fall risk among established unilateral lower limb prosthesis users: cutoff scores and associated validity indices. PM R.

[17] Maitland ME, Allyn KJ, Ficanha E, Colvin JM, Wernke MM (2020). Finite element simulation of prosthetic foot adaptation to mediolateral-angled cross-slopes. J Prosthet Orthot.

