# Supplementary material for: The Effectiveness of Frontal Plane Adaptability in a Novel Foot Prosthesis: Protocol for a Randomized Crossover Clinical Trial
Source: JMIR Res Protoc. 2025 Dec 23;14:e84313. doi: 10.2196/84313 (PMC12724065; doi:10.2196/84313)
Supplement: Peer Review Report 1 [file resprot-v14-e84313-s001.pdf]

**Department of Defense**
**U.S. Army Medical Research and Development Command**
**Congressionally Directed Medical Research Programs**
**Fiscal Year 2022 Orthotics and Prosthetics Outcomes Research Program**
**Clinical Trial Award - Funding Level 2**
**Peer Review Summary Statement**

**CDMRP Log Number:** OP220033
**Grants.gov ID Number:** GRANT13626556
**Review Panel:** Clinical Trial
**Discussion Period:** 08/29/2022-08/30/2022

**Project Duration:** 48 months
**Budget Requested:** $2,610,721
**Direct Costs:** $2,183,616
**Indirect Costs:** $427,105

**Title:** The Effectiveness of Frontal Plane Adaptability in a Novel Foot Prosthesis for People With Above-Knee Amputations, Bilateral Amputations, or Limited Mobility
**Principal Investigator:** Murray Maitland
**Performing Organization:** Washington, University of
**Contracting Organization:** Washington, University of

## OVERVIEW

The Principal Investigator (PI) of this application proposes to evaluate the effects of a frontal plane adaptable linkage on mobility, socket comfort, performance, and activities of daily living for people with above-knee amputations, bilateral amputations, or below-knee amputations and limited mobility. The specific aims are (1) in the primary analysis, to compare locked and unlocked conditions of the linkage system, and in the secondary analysis, to compare the unlocked version of the investigational foot and the person's usual foot (standard of care); and (2) to examine the participant's lived experience during community mobility through qualitative analysis of a logbook and interviews. A randomized crossover clinical trial design following an A1-B1-B2-A2 format will be performed. The interventions are the participant's usual prosthetic foot and locked or unlocked frontal plane adaptable linkages. The projected results are improved performance on the 10-Meter Walk Test, the Timed Up and Go Test, the Three Times Figure of Eight Walk Test (3XF8W), and the Four-Square Step Test (FSST) as well as the Prosthetic Limb Users Survey of Mobility (PLUS-M) questionnaire to measure participants' perception of physical function, mobility, and balance.

*Procurement Sensitive Document*
*Do not copy or distribute without CDMRP written permission.*
*Prepared by SRA International, Inc., A GDIT Company, a support contractor to CDMRP*

| | Average Score | Standard Deviation |
|---|---|---|
| **Overall Evaluation**<br>*Rating Scale: 1.0 (highest merit) to 5.0 (lowest merit)* | 1.7<br>**(Excellent)** | 0.2 |
| | | |
| **Criteria**<br>*Rating Scale: 10 (highest merit) to 1 (lowest merit)* | **Average Score** | |
| **Impact** | 8.0 | |
| **Research Strategy and Feasibility** | 7.2 | |
| **Intervention** | 8.2 | |
| **Regulatory Strategy and Transition Plan** | 9.0 | |
| **Recruitment, Accrual, and Feasibility** | 7.0 | |
| **Statistical Plan and Data Analysis** | 7.6 | |
| **Ethical Considerations** | 8.7 | |
| **Personnel and Communication** | 8.0 | |

## SCORED CRITERIA

***Impact***
Average Score: 8.0

**Scientist Reviewer A**

Strengths: The proposed research applies to the FY22 OPORP strategic focus of prosthetic technology prescription. If successful, the results of this study will help to optimize patient-specific technology prescription for the patients with limb loss and mobility deficits. More specifically, this project focuses on individuals with above-knee amputations, bilateral amputations, or below-knee amputations and limited mobility who often do not have the option to receive prosthetic feet with frontal plane flexibility. The findings from this study will shed light on a current controversy regarding whether people with lower levels of mobility can benefit from more complex feet, such as those that can move and flex in different directions which may help influence standard of care and Centers for Medicare & Medicaid Services policy.

Weaknesses: The proposed study will only collect short-term outcomes (2 weeks) concerning activities of daily living but will not determine whether long-term use of the proposed prosthetic foot is associated with any benefits or potential harm. Although it is indicated that findings from this study may allow for reduced time to return to duty in military service members, lack of collaboration with a DOD partner may limit achieving this goal.

**Scientist Reviewer B**

Strengths: This proposal addresses the strategic goal to optimize patient-specific technology prescription with device characteristics and, to a lesser amount, human interface with devices. The improvement of prosthetic feet in the ability to safely walk on uneven and sloped terrain will greatly help all amputees, especially active military.

Weaknesses: No weaknesses were noted.

**Consumer Reviewer**

Strengths: For purposes of enabling this product to be available across a wider spectrum of lower limb amputees, this study will serve reaching that goal. The market should enable this product to be available.

Weaknesses: The abstract is a bit self-defeating; the META is currently a high-activity foot. Service members who suffer a blast injury and amputation are high-activity individuals; thus the META would serve that need. Similar designs are on the market for K1/K2 activity level from OttoBock and Ossur, reimbursement codes notwithstanding. The need for the study is not fully supported.

***Research Strategy and Feasibility***
Average Score: 7.2

**Scientist Reviewer A**

The proposed study design is a multisite, randomized crossover clinical trial comparing a carbon fiber foot that includes a functional, unlocked frontal plane adaptable linkage to the same foot with the linkage locked to prevent frontal plane motion. With the linkage locked, the foot is comparable to energy storage and return of a carbon fiber foot. The secondary aim for this clinical trial compares the unlocked foot to the participant's usual foot, representative of the usual standard of care. The testing will be conducted following an A1-B1-B2-A2 design. The A conditions are focused on the participant's usual prosthetic foot. The B conditions are focused on locked and unlocked frontal plane adaptable linkages with the order randomized. Block randomization, stratified by study site, will be used to determine the order participants receive the locked and unlocked prosthetic. A simple pin system has been developed to prevent the linkage from rotating for the study.

Strengths: The scientific rationale for the proposed clinical trial is appropriately supported by the preliminary data, critical review, and analysis of the literature in the application. The crossover design of the study is appropriate for the study aims, and the study population is representative of the target population that might benefit from the findings of the study. The application clearly identifies the level of evidence that will result from the proposed work, and it is consistent with the study research design.

Weaknesses: Inclusion/exclusion for group assignments and the characteristics of each group are not particularly described well in the narrative. It is unclear why the recruitment target will include an unequal number of the 3 different patient categories mentioned in the application: (1) people with unilateral above-knee amputations (n = 40), (2) people with bilateral lower extremity amputations (n = 16), and (3) people with unilateral below-knee amputations (n = 40). The proposed outcome measures appear to be appropriate, but it is unclear which outcome measure will be evaluated to indicate success in the study as there are currently 3 main outcomes included in the analysis. It is unclear if the study would be deemed successful if only 1 of 3 primary outcomes indicates significantly positive scores for the study device.

**Scientist Reviewer B**

Strengths: The preliminary work supports the premise of the proposed trials very well. The randomized crossover design is appropriate. The evaluations are good and include both subjective data (Prosthetic Limb Users Survey of Mobility [PLUS-M], logbooks, and semistructured interviews) as well as performance tests (10-Meter Walk Test, Timed Up and Go, 3 times figure 8, Four-Square Step, and gait analysis with a Zeno Walkway). The potential problems are described. The primary issue would be recruitment and retention. These issues are addressed, and mitigation is described. The number of subjects

*Procurement Sensitive Document*
*Do not copy or distribute without CDMRP written permission.*

seems low in each group, but it does fit the power analysis, and the recruitment issues are addressed. The design of the research should enable clear results of the effectiveness of the foot design.

Weaknesses: There is no washout period between the interventions: locked and unlocked conditions. This may not be an issue for this type of trial, but it is not addressed. All of the assessments are walking on level ground. Flexible and adaptive feet have been shown to be useful for walking on uneven terrain and for improving balance. It would be very useful to include these assessments with this foot. The period of use, 2 weeks, is short. This may not permit full adaption to the feet, and assessments in daily living.

***Intervention***
Average Score: 8.2

**Scientist Reviewer A**

Strengths: A licensed prosthetist will fit each participant with the prosthetic foot. The prosthetic foot is commercially available which reduces potential regulatory issues and will be available for all participants for the duration of the proposed clinical trial. The proposed intervention directly addresses the clinical needs of the patient population described in the application. Clinical evidence to support the safety and stability of the intervention has been evaluated in a group of higher-functioning patients with limb loss.

Weaknesses: There is no information provided on whether a 2-week phase is adequate for patients to notice a significant difference in their activities as suggested in the proposal. Since there are at least a couple of similar products available in the market, it is not clear what the advantage of the prosthetic foot is over the comparators for the purposes of this application.

**Scientist Reviewer B**

Strengths: The intervention to be studied is the improvement in mobility, safety, and walking abilities with a pronating/supinating foot. It is compared with the standard of care: fixed foot. The same foot is used in a locked or unlocked condition, and the subjects will use their current feet and socket, so the conditions of the 2 cases will be the same except for the locked and unlocked conditions. This eliminates confounding conditions.

Weaknesses: No weaknesses were noted.

***Regulatory Strategy and Transition Plan***
Average Score: 9.0

**Scientist Reviewer A**

Strengths: The application identifies that the University of Washington (UW) has intellectual property protection for the investigational foot and that the PI is the primary inventor listed on all patents. The product is categorized as a Class I medical device by the FDA and does not require clearance. At the conclusion of this project, there will be no direct products to commercialize since the prosthetic foot technology was commercialized in 2020. However, application of these findings may lead to future developments to further enhance the biomimetic design of other components. The focus of the transition plan will be to disseminate the research findings for use in guiding clinical practice and policy changes. The milestones for incorporation of the findings into clinical practice and future funding opportunities are realistic, reasonable, and achievable.

*Procurement Sensitive Document*
*Do not copy or distribute without CDMRP written permission.*

Weaknesses: There are no plans for engaging individuals with limb loss as stakeholders in the transition plan. No other weaknesses were noted.

**Scientist Reviewer B**

Strengths: The META Arc foot that will be studied is a Class I device by the FDA. There are no regulatory issues. WillowWood and UW have a joint patent agreement. The industry partner, WillowWood, is already involved.

Weaknesses: No weaknesses were noted.

**Technology Transfer Specialist Reviewer**

Strengths: The applicant submitted documentation supporting the exemption of the product. The strategy and milestones seem achievable. There is strong collaboration listed in support of the distribution of the knowledge product. Intellectual property is well established, and the applicant does not plan on future IP. This application has little explicit risk.

Weaknesses: No weaknesses were noted.

***Recruitment, Accrual, and Feasibility***
Average Score: 7.0

**Scientist Reviewer A**

Strengths: The study team has been successful in the past in recruiting for similar studies for patients with higher level of mobility, and a multifaceted recruitment strategy has been proposed for the study.

Weaknesses: The overall availability of the targeted population at the study sites is not provided, making it unclear whether target enrollment of 96 individuals with mobility restrictions will be feasible. The anticipated dropout rate of 12% appears to be overoptimistic and could have significant consequences. It is unclear why the recruitment target will include unequal numbers of the 3 different patient categories mentioned in the application: (1) people with unilateral above-knee amputations (n = 40), (2) people with bilateral lower extremity amputations (n = 16), and (3) people with unilateral below-knee amputations (n = 40).

**Scientist Reviewer B**

Strengths: Recruitment and attrition are listed as the main issues in the proposal. These are addressed. The informed consent is appropriate. The inclusion and exclusion criteria are appropriate. A plan for the recruitment of minorities and women is given with desired numbers or recruits.

Weaknesses: Recruitment and attrition are the primary difficulties in the proposed work. There is no contingency plan if the goals are not met.

***Statistical Plan and Data Analysis***
Average Score: 7.6

**Scientist Reviewer A**

Strengths: Overall the statistical analysis plan seems appropriate for the proposed study. A sample size calculation has been included. The study team includes a biostatistician and a qualitative researcher. Planned subgroup analyses have been included to evaluate the differences in gender and ethnicity.

Weaknesses: The trial is powered for statistical significance, but the numbers of participants in each group are not large by clinical trial standards. There are 3 outcomes suggested for the primary aim (time to complete the FSST and time and number of steps to complete the 3XF8W) at a 2-sided 0.05 significance level. It is unclear how the findings from each outcome will be used to assess the success of the overall study. For example, it is unclear if the trial will be deemed successful if only 1 out of 3 analyses proves to be significant. There are several references made to having "good power" in the sample size section, but it is unclear what that means within the context of the proposed study. It is unclear how the heterogeneity in the sample will be evaluated statistically as there are 3 proposed groups with different sample sizes that could respond very differently to the intervention.

**Scientist Reviewer B**

Strengths: Normality and homogeneity are to be checked. The multivariant linear mixed-effects model is appropriate if the normality and homogeneity conditions are met.

Weaknesses: The methods of checking normality and homogeneity are not given. No alternative analysis methods are listed if the normality and homogeneity conditions are not met.

**Biostatistician Reviewer**

Strengths: Both primary and secondary outcome measures are clearly defined, in line with the study objectives. Multivariate linear mixed model is appropriate for the study with clear specification of fixed and random effects. Proposed sample sizes are justified with clearly defined effect size and the target value derived from previous studies on PLUS-M scores.

Weaknesses: It is a bit concerning that the method to avoid and/or justify carryover effect is not adequately discussed. Toward this end, a washout period may be helpful in between different treatment periods. This issue persists when comparing the unlocked condition with usual care. Power analysis related to the primary performance measures is not adequately provided. It is not clear whether multiplicity adjustment will be performed as there are both performance and satisfaction measures.

***Ethical Considerations***
Average Score: 8.7

**Scientist Reviewer A**

Strengths: The target population selected for the proposed trial stands to significantly benefit from the knowledge gained. The level of risk to human subjects is minimal in this study, and the safety monitoring and reporting plan is appropriate for the level of risk. A detailed plan to protect privacy and confidentiality of participants is provided. An informed consent process is also described and appears to be appropriate for the study.

Weaknesses: No weaknesses were noted.

*Procurement Sensitive Document*
*Do not copy or distribute without CDMRP written permission.*

**Scientist Reviewer B**

Strengths: There are no issues with ethical concerns. Potential risks and benefits are clearly given. All privacy issues are addressed. The data will be anonymized.

Weaknesses: There are potential financial conflicts of interest for key personnel that are not detailed in the informed consent.

**Bioethicist Reviewer**

Strengths: The study design is a randomized crossover clinical trial, which includes individuals with above-knee amputations, bilateral amputations, or unilateral below-knee amputations who have lower levels of mobility. These are the individuals who may benefit from participating in the research and will benefit from the results of the research. The expected risks seem reasonable given the possible benefits of improving mobility and reducing socket forces during activities of daily living. The recruitment process protects the privacy of the individual enrolling. The data management strategy is sufficient to protect the confidentiality of the data provided.

Weaknesses: The submission indicates that home visits might occur. They need to specify the protocol for home visits. .Given that this is submitted under FL2, as a large pragmatic, randomized, comparative effectiveness trial, this should account for limited-English or non-English speaking individuals. The application discusses enrollment of a diverse population, but individuals must speak and understand English to participate. The consent form includes video of performance/functional tasks and motion analysis, but this is not addressed elsewhere regarding privacy, only that the interviews would be recorded and transcribed.

***Personnel and Communication***
Average Score: 8.0

**Scientist Reviewer A**

The PI, Murray Maitland, received a PhD degree in joint injuries and arthritis from the University of Calgary in 1996 and is associate professor of rehabilitation medicine at UW.

Coinvestigator Donald Fogelberg received a PhD degree in occupational science from the University of Southern California in 2008 and is associate professor of rehabilitation medicine at UW.

Coinvestigator Andrew Humbert received a PhD degree in biostatistics from UW in 2020 and is assistant professor of rehabilitation medicine at UW.

Site PI James Colvin received an MS degree in biomedical engineering from The Ohio State University in 1986 and is director of research and technology at WillowWood Global, LLC.

Site PI Matthew Wernke received a PhD degree in biomedical engineering from the University of South Florida in 2013 and is research and development manager at WillowWood.

Site PI Stephanie Carey received a PhD degree in biomedical engineering from the University of South Florida in 2008 and is research professor of mechanical engineering at the University of South Florida.

Murray Maitland, PT, PhD, is an associate professor in the Department of Rehabilitation Medicine at the University of Washington

Donald Fogelberg, OT, PhD, is an associate professor within the Department of Rehabilitation Medicine at the University of Washington

Strengths: Murray Maitland, PT, PhD, is trained as a clinical physical therapist and research biomechanist. He has designed, created, and studied prototype prosthetic feet. Dr Maitland will lead the oversight, planning, management, and evaluation of the progress of the project. Donald Fogelberg, OT, PhD, has expertise in qualitative research methods, patient-reported outcomes, and questionnaire development. Dr Fogelberg will conduct semistructured interviews and will oversee the analysis of qualitative information. Andrew Humbert, PhD, is a biostatistician and has 8 years of experience in study design and data analysis in medical studies using various research designs. Dr Humbert will assist with study design, statistical data analysis and interpretation, and preparation of manuscripts for dissemination. This is a strong multidisciplinary team with appropriate expertise and experience in the domains proposed for the study. A dedicated biostatistician with clinical trials experience is included with the study team. The levels of effort of the study team members are appropriate for successful conduct of the proposed trial.

Weaknesses: The multi-institutional structure governing the research protocol(s) across all participating institutions is not well described. The level and frequency of communication between sites is not delineated.

**Scientist Reviewer B**

Strengths: The team has a history of prosthetic design and research. It is a strong team. Designers, biomechanics researchers, and statisticians create a strong team.

Weaknesses: There are 3 sites for the work: University of Washington, WillowWood, and University of South Florida. Though the team has worked together, a coordination plan is not adequately provided in the proposal.

## UNSCORED CRITERIA

***Environment***

**Scientist Reviewer A**

The scientific environment, the clinical setting, and the accessibility of institutional resources to support the clinical trial at each participating institution appear appropriate. Letters of institutional support and collaboration are included with the application.

**Scientist Reviewer B**

The device to be evaluated, the META Arc foot, is manufactured by WillowWood, so there are no concerns with the availability of the test devices. All equipment and facilities are excellent.

***Budget***

**Scientist Reviewer A**

The proposed budget for the study appears appropriate with adequate levels of effort included. Funds for participant reimbursement and travel are appropriately included. Tuition support for a graduate student and funds for a research coordinator are also requested which are appropriate.

**Scientist Reviewer B**

The budget is appropriate for the research.

***Application Presentation***

**Scientist Reviewer A**

There were excessive white spaces at the bottom of several pages of the narrative that were distracting. Otherwise, the proposal was well written, and the information was presented appropriately.

**Scientist Reviewer B**

The application is well written. It would be good to show the foot kinematics clearer. It is difficult to see the kinematics in the photos. A kinematic diagram showing the instant center would help.

*Procurement Sensitive Document*
*Do not copy or distribute without CDMRP written permission.*
